# Benchmarking the generalizability of brain age models: Challenges posed by scanner variance and prediction bias

**DOI:** 10.1002/hbm.26144

**Published:** 2022-11-08

**Authors:** Robert J. Jirsaraie, Tobias Kaufmann, Vishnu Bashyam, Guray Erus, Joan L. Luby, Lars T. Westlye, Christos Davatzikos, Deanna M. Barch, Aristeidis Sotiras

**Affiliations:** ^1^ Division of Computational and Data Sciences Washington University in St. Louis St. Louis Missouri USA; ^2^ Department of Psychiatry and Psychotherapy, Tübingen Center for Mental Health University of Tübingen Tübingen Germany; ^3^ Norwegian Centre for Mental Disorders Research, Division of Mental Health and Addiction, Oslo University Hospital & Institute of Clinical Medicine University of Oslo Oslo Norway; ^4^ Center for Biomedical Image Computing and Analytics, Department of Radiology University of Pennsylvania Philadelphia Pennsylvania USA; ^5^ Department of Psychiatry Washington University in St. Louis St. Louis Missouri USA; ^6^ Department of Psychology University of Oslo Oslo Norway; ^7^ KG Jebsen Centre for Neurodevelopmental Disorders University of Oslo Oslo Norway; ^8^ Department of Psychological & Brain Sciences Washington University in St. Louis St. Louis Missouri USA; ^9^ Department of Radiology Washington University School of Medicine in St. Louis St. Louis Missouri USA

**Keywords:** brain age, brain development, computational neuroscience, generalizability, machine learning

## Abstract

Machine learning has been increasingly applied to neuroimaging data to predict age, deriving a personalized biomarker with potential clinical applications. The scientific and clinical value of these models depends on their applicability to independently acquired scans from diverse sources. Accordingly, we evaluated the generalizability of two brain age models that were trained across the lifespan by applying them to three distinct early‐life samples with participants aged 8–22 years. These models were chosen based on the size and diversity of their training data, but they also differed greatly in their processing methods and predictive algorithms. Specifically, one brain age model was built by applying gradient tree boosting (GTB) to extracted features of cortical thickness, surface area, and brain volume. The other model applied a 2D convolutional neural network (DBN) to minimally preprocessed slices of T1‐weighted scans. Additional model variants were created to understand how generalizability changed when each model was trained with data that became more similar to the test samples in terms of age and acquisition protocols. Our results illustrated numerous trade‐offs. The GTB predictions were relatively more accurate overall and yielded more reliable predictions when applied to lower quality scans. In contrast, the DBN displayed the most utility in detecting associations between brain age gaps and cognitive functioning. Broadly speaking, the largest limitations affecting generalizability were acquisition protocol differences and biased brain age estimates. If such confounds could eventually be removed without post‐hoc corrections, brain age predictions may have greater utility as personalized biomarkers of healthy aging.

## Graphical abstract

We examined the out‐of‐sample predictions of previously developed brain age models, which were chosen based on the size and diversity of their training data. Broadly speaking, no single model was uniformly better across all test samples or facets of generalizability. The largest limitations affecting generalizability were scanner‐related variance and biased brain age predictions.

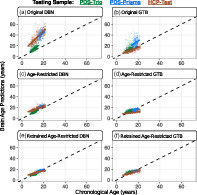

## 1 INTRODUCTION

Establishing growth charts of normative brain development has been a longstanding objective for the neuroscience community. A standardized biomarker of brain development would have immense utility for identifying risks of adverse psychological outcomes, which may lead to more precise and personalized interventions. One approach toward establishing growth charts has been to leverage machine learning models that estimate the biological age of a person based on their brain structure or connectivity (i.e., brain age). Prior research using magnetic resonance imaging (MRI) has shown that brain age predictions can be fairly accurate when applying deep learning techniques (Kuo et al., 2021; Leonardsen et al., 2022), or more traditional machine learning methods (Cole & Franke, 2017), to multiple types of imaging modalities (Schulz et al., 2020) and derived brain features (Chen et al., 2020). Many studies also underscore how deviations between brain and chronological ages (i.e., brain age gaps) are useful for differentiating individuals as a function of cognitive impairment, psychopathology, and neurodegenerative disorders (Franke & Gaser, 2012; Gaser et al., 2013; Liem et al., 2017). Furthermore, longitudinal studies have demonstrated that brain age models can be reliable across time even when applied to clinical samples (Høgestøl et al., 2019; Richard et al., 2020). A few models from these studies were made publicly available with the intention that they would be applicable in novel research and clinical settings, yet systematic tests of generalizability are sparse.

The current study examined the out‐of‐sample predictions from two brain age models, which were chosen based on the size and diversity of their training data. One brain age model, termed as the Deep Brain Network (Bashyam et al., 2020) (DBN), used a 2D convolutional neural network that makes predictions from axial slices of T1‐weighted images. The second model used gradient tree boosting (Kaufmann et al., 2019) (GTB) to compute sex‐specific predictions based on extracted features of brain volume, surface area, and cortical thickness from T1‐weighted scans. Both models were developed and evaluated using large training and test samples (DBN: 17,410; GTB: 45,615), which were aggregated across many sites and scanner types and comprised individuals from 3 to 95 years of age. The published results from both studies demonstrated that brain age predictions were accurate when applied to many healthy developing subsamples and useful for differentiating between groups that exhibited different forms of pathology. The predictive power of both models was established using cross‐validation, demonstrating that the models were generalizable across sites, sexes, and developmental stages (Bashyam et al., 2020; Kaufmann et al., 2019). Taken together, these two brain age models appear to have the most potential for becoming useful growth charts of brain development.

Despite the strengths of these brain age models, several challenges may hinder their utility. First, sampling biases can occur when characteristics of the training sample (e.g., age, sex, health status, etc.) are not evenly distributed across age bins (de Lange et al., 2022). Second, brain age gaps tend to regress towards the mean of their training sample, such that predictions for younger participants are more likely to be overestimated, while the ones for older participants are underestimated (Liang et al., 2019). Lastly, MRI scan properties may vary significantly due to differences in scanner types and acquisition parameters (Han et al., 2006; Jovicich et al., 2009), which can lead to biases when machine learning models are not trained and tested on balanced samples collected with similar protocols (Jonsson et al., 2019; Liem et al., 2017). The best way to account for these limitations is under debate (Butler et al., 2021), as such we evaluated the potential strengthens and challenges of each model under a variety of conditions.

The generalizability of the DBN and GTB was assessed by applying them to three diverse early‐developing cohorts (Luby et al., 2010; Somerville et al., 2018) that included both cross‐sectional and longitudinal data from different scanner types and acquisitions protocols. Each model was applied to scans with varying levels of image resolution, gray/white matter contrast, and signal‐to‐noise ratios (Magnotta et al., 2006; Sadri et al., 2020). Furthermore, a multi‐faceted approach was taken to understand generalizability in terms of accuracy, reliability, and utility to detect individual differences in cognition. The differing sampling characteristics and acquisition parameters of our three cohorts may provide a stronger test of generalizability, despite their sample sizes being relatively small and not encompassing the full lifespan. These additional tests are essential for assessing a model's capacity to provide meaningful predictions in clinical settings, where sampling and imaging properties might vary considerably.

Based on prior machine learning (Poldrack et al., 2020; Varoquaux et al., 2017) and brain age studies (de Lange et al., 2022; Liem et al., 2017), we hypothesized that the out‐of‐sample predictions from both models would not be as accurate compared to their cross‐validation results (Bashyam et al., 2020; Kaufmann et al., 2019). Specifically, we predicted that the models would encounter challenges due to sampling‐bias, scanner variance, and prediction bias, which may result in systematic over or underestimations in brain age predictions. As such, we created additional variants of the DBN and GTB to understand how model performance changed when their training samples became more similar to our testing samples. Accordingly, a second set of the models was trained only using youths between the ages of 8 to 21 that were part of its original sample (see methods: “age‐restricted models”). Since the training samples for the age‐restricted models primarily consisted of Siemens Trio scans, another set of model variants was created by retraining the age‐restricted models with Siemens Prisma scans (see methods: “retrained age‐restricted models”). Given prior generalizability findings (Liem et al., 2017), we predicted that the retrained variants would yield the most accurate brain age estimates. To the best of our knowledge, there were not any prior studies to inform our hypotheses of model reliability or utility to detect differences in cognition.

## 2 METHODS AND MATERIALS

### 2.1 Overview of testing data sets

Three youth cohorts were utilized to ensure our findings would generalize across different sampling characteristics and acquisition parameters. These data sets were gathered from the Preschool Depression Study (Luby et al., 2010) (PDS) and Human Connectome Project in Development (Somerville et al., 2018) (HCP‐D). The HCP‐D is a cross‐sectional multi‐site study consisting of 789 participants that underwent MRI scanning and cognitive assessments. As discussed further below, half of the HCP‐D sample was used to retrain each of the brain age models so generalizability could be assessed as the training and testing data became more similar. The remaining half (n = 394) was used to evaluate the generalizability of all brain age models (HCP‐Test). The split‐half procedure assured that both groups would be matched by age at scan, sex, and image quality metrics (i.e., Euler Number Rosen et al., 2018). The PDS is a 5‐wave neuroimaging sample consisting of 167 participants who completed cognitive assessments in the final two waves. The first three waves of the PDS were completed with a Siemens TIM Trio scanner (sessions: 432), whereas the final two waves used a Siemens Prisma (sessions: 280). Given the differences in scanner types and availability of cognitive data, the two sets of PDS data were treated as different studies with the three waves referred to as PDS‐Trio and the final 2 waves as PDS‐Prisma. Distributions of age differed across these three samples (Table 1), but all participants were youths between the ages of 8–22 years (Figure S1).

**TABLE 1:** Sample characteristics and image quality metrics

Variables of interest	PDS‐Trio	PDS‐Prisma	HCP‐Test	*F*‐value	*p*‐value
Age in Years	11.6 (1.7)	17.4 (1.5)	13.8 (4.1)	376.1	<0.001
Working Memory	–	104.2 (16)	105.6 (14)	1.5	0.23
Processing Speed	–	97.9 (22)	105.4 (22)	18.1	<0.001
Episodic Memory	–	104.5 (18)	106.2 (17)	1.4	0.25
Vocabulary	–	105.6 (17)	112.1 (16)	24.3	<0.001
Cognitive flexibility	–	90.5 (15)	92.3 (14)	3.8	0.05
Euler number	−195.3 (152)	−41.5 (25)	−41.2 (18)	333.2	<0.001
Contrast‐To‐Noise ratio	249.6 (804.1)	269.5 (702.4)	15.9 (4.6)	19.7	<0.001
Peak Signal‐To‐Noise Ratio	10.5 (0.4)	10.61 (0.4)	13.3 (0.6)	5107.0	<0.001
Mean Signal‐To‐Noise Ratio (1–4)	81.9 (208.6)	93.8 (242.9)	15.6 (3.4)	26.3	<0.001
Mean foreground intensity values	159.9 (22.9)	568.9 (53.9)	486.9 (70.7)	6504.0	<0.001

*Note*: Descriptive statistics are reported for each numerical variable from both subsets of the PDS and the HCP‐Test. The first three columns denote the sample mean and standard deviation in parentheses. One‐way ANOVAs demonstrated that the test samples differed across most sample characteristics and all image quality metrics. There were slightly more males than females for the PDS (males = 87; females = 80) as well as the HCP sample (males = 414; females = 411).

### 2.2 Cognitive phenotyping

The NIH‐Toolbox Cognition Battery (Weintraub et al., 2013) was completed as part of the PDS‐Prisma and HCP‐D studies. Individual differences were assessed across the following five cognitive domains. Working memory was quantified using the List Sorting test. Processing speed was assessed through the Pattern Comparison Test. Episodic memory was measured by the Picture Sequencing test. Language was measured by the Picture Vocabulary test. Attention was captured through the Flanker Inhibitory Control test. All analyses herein used the age‐adjusted t‐scores that were produced by NIH‐Toolbox (Casaletto et al., 2015).

### 2.3 Imaging acquisition

The PDS‐Trio was the only sample that was scanned on a 3 T Siemens TIM Trio with a 12‐channel head coil. These magnetization‐prepared, rapid acquisition gradient‐echo (MPRAGE) T1‐weighted images had the following acquisition parameters: 1 mm isotropic resolution; TR 2.4 ms; TE 3.16 ms; 160 sagittal slices; flip angle 8°; FOV 256 × 256 × 224 mm^3^; 6:18 acquisition time. The PDS‐Prisma utilized a 3 T Siemens Prisma with a 32‐channel head coil, but the acquisition parameters were identical to those from the PDS‐Trio, except that the TE was lowered from 3.16 to 2.22 ms and the acquisition time was 20 seconds longer. Lastly, the HCP‐D was also scanned on a 3 T Siemens Prisma with a 32‐channel head coil. However, the HCP acquisition parameters were further optimized to enhance image quality: 0.8 mm isotropic resolution; TR 2.4 ms; TE 2.14 ms; 208 sagittal slices; flip angle 8°; FOV 320 × 320 × 300 mm^3^; 6:54 acquisition time. This protocol also included embedded volumetric navigators (vNavs) to correct for in‐scanner head motion and minimize the impact of such artifacts (Harms et al., 2018).

### 2.4 Preprocessing pipelines and quality assurance

All three test samples were processed using the exact procedures described in the research articles that computed the original brain age models (de Lange et al., 2022; Kaufmann et al., 2019). Briefly, the DBN utilized a preprocessing procedure that involved bias correction, multi‐atlas skull stripping (Doshi et al., 2013) using six templates that were derived from the Philadelphia Neurodevelopmental Cohort (Satterthwaite et al., 2014) (PNC) and linear registration (Jenkinson et al., 2002) to the 1 mm MNI‐152 template (Fonov et al., 2011). The resulting scans were divided into 80 horizontal slices before applying the 2D‐convolutional neural network, which generated a brain age prediction for each slice. The median prediction was taken to represent the final brain age for a given scan. All brain age estimates were derived from a signal model, irrespective of sex‐differences in brain development.

In preparation for the GTB, scans underwent automated surface‐based morphometry and subcortical segmentation using FreeSurfer (Fischl, 2012) version 5.3. Neuroimaging features were extracted from the multimodal Glasser 2016 atlas (Glasser et al., 2016), which comprised 180 regions per hemisphere. Altogether, the GTB predictions were based on 1118 neuroimaging features consisting of cortical thickness, surface area, cortical volume, and subcortical volume. Sex‐specific models were used to account for neurodevelopmental differences between males and females.

A trained MRI analyst performed visual inspections of the raw T1‐weighted images to ensure that scans with substantial artifacts were excluded prior to this study. To account for individual differences in image quality, the Euler number from FreeSurfer was included as a covariate for all analyses. Previous studies have demonstrated that the Euler number can be a proxy for visual ratings of image quality (Kaufmann et al., 2019; Rosen et al., 2018), though its original purpose is to summarize the topological complexity of the reconstructed cortical surface (Dale et al., 1999). Sensitivity analyses within the HCP‐Test sample were performed by also covarying for individual differences in image quality through the vNavs measure, which represents the number of re‐acquired slices due to head movement during acquisition.

### 2.5 Original brain age models (DBN / GTB)

Three variants of the DBN and GTB were analyzed to better determine how performance changed based on alterations in their training data; six brain age model were evaluated altogether. The first set of brain age models was applied without any changes to the training data. As described in the published articles (Bashyam et al., 2020; Kaufmann et al., 2019), each of the original models benefited from data aggregation methods to obtain large training samples (DBN = 11,729; GTB = 35,474) that covered the lifespan (DBN = 3–95; GTB = 3–96). Both models were optimized on their respective training data using fivefold cross validation, which resulted in robust correlations between chronological and predicted brain ages (DBN = 0.978; GTB = 0.935).

### 2.6 Age‐restricted brain age models (rDBN / rGTB)

Given that the original models were trained across the lifespan, it could be problematic that the testing samples herein pertained exclusively to youths between the ages of 8 and 22 years. This concern led to the creation of a second set of DBN and GTB models, where the age range of the training data was restricted to only include scans that overlapped in age with the test samples. The resulting age‐restricted training data was substantially reduced in size (rDBN = 1794; rGTB = 3382) and primarily consisted of scans from the PNC (Satterthwaite et al., 2014) and the pediatric imaging, neurocognition and genetics (Jernigan et al., 2016) (PING) studies. These age‐restricted model variants were computed using the identical procedures and software packages described in the original research studies (Bashyam et al., 2020; Kaufmann et al., 2019).

### 2.7 Retrained age‐restricted brain age models (tDBN / tGTB)

A potential concern for the age‐restricted model variations was regarding scanner type since the majority of PNC and PING data was collected using Siemens TIM Trio scanners and few were acquired from Siemens Prisma. To minimize the impact of potential scanner‐related variance, the age‐restricted model variants were retrained by including additional Siemens Prisma scans from the HCP‐D. Retraining was completed using the entire HCP‐D sample (n = 789) when evaluating generalizability among the PDS‐Trio and PDS‐Prisma scans. Retraining was repeated with only half of the HCP‐D data (n = 395), such that generalizability of all model variations could be assessed on the remaining HCP‐Test sample. The DBN underwent transfer learning by using the new scans to update the model weights. Only the last layer of the DBN was unfrozen for the initial epoch, but the following 28 epochs were performed with all layers unfrozen. Given that it is not possible for gradient boosting models to undergo transfer learning, the GTB model was retrained from scratch by combining the novel HCP‐D scans with the age‐restricted training data.

### 2.8 Statistical analysis of model accuracy

The accuracy of each brain age model was evaluated by the linear fit between brain and chronological ages, deriving the following metrics: slope, y‐intercept, mean absolute error (MAE), and Pearson correlation. The slopes and y‐intercepts are useful metrics for quantifying potential prediction biases or scaling effects, which reflect systematic over or underestimations in brain age predictions (Butler et al., 2021). Theoretically, prediction bias and scaling effects would be less substantial as slopes approach one and y‐intercepts approach zero. The goodness of fit for a given model improves as MAEs approach zero, though recent evidence suggests that moderately‐fit models (MAE: 3–6 years) are most useful for detecting individual differences (Bashyam et al., 2020). It is worth noting that correlations weaken when the age range of the test sample is restricted (Poldrack et al., 2020), thereby limiting our ability to make comparisons with prior studies and between the HCP‐Test (range: 16.6 years) and the PDS subsamples (Trio: 8.3 years; Prisma: 8.2 years). Given these challenges, we primarily assessed the goodness of fit for a model based on the prediction errors as opposed to Pearson correlation. Analyses of model accuracy were performed on raw brain age predictions, though supplemental analyses were conducted on “corrected” brain age predictions that underwent a post‐hoc adjustment to remove any potential bias in brain age predictions (Smith et al., 2019).

### 2.9 Statistical analysis of model reliability

Model reliability was assessed to investigate the consistency of both machine learning frameworks when applied to each test sample. As such, we quantified the degree of variability across the original, age‐restricted, and retrained variants of the GTB and DBN respectively. Deviation scores were computed by min/max scaling the brain age gaps for each variant and subsequently calculating the standard deviation from all three variants of the DBN and GTB on an individual basis (e.g., a deviation value for each scan). Generalized additive models were used to further evaluate whether individual differences in deviation scores could be explained by age, sex‐differences, or image quality. To understand the potential confounding influence of prediction bias on model reliability, supplemental analyses were conducted using the “corrected” brain age gaps that underwent a post‐hoc adjustment so that they would be orthogonal with chronological age (Smith et al., 2019).

### 2.10 Statistical analysis of model utility

Model utility was operationalized by measuring each model's ability to detect individual differences in cognition. The raw brain age prediction was always the response variable, and the main predictor was an age‐adjusted score for a given cognitive domain, while covarying for chronological age, Euler number, and sex. Given the co‐linearity between age and image quality in youth (Rosen et al., 2018), these models were chosen to ensure that the brain age gaps would be orthogonal to head motion confounds and prediction bias. All models herein used linear regressions for the HCP‐Test data and mixed effect models with random intercepts for each participant in the PDS‐Trio and PDS‐Prisma data sets. Analyses were performed using R version 4.0.2 (Team R. C, 2013). All code and model variants pertaining to this study have been made available through the following GitHub repository: https://github.com/ccplabwustl/RobertJirsaraie/tree/master/proj20-BrainAgeEval.

## 3 RESULTS

### 3.1 Model accuracy: How similar are chronological and predicted brain ages?

**FIGURE 1:**
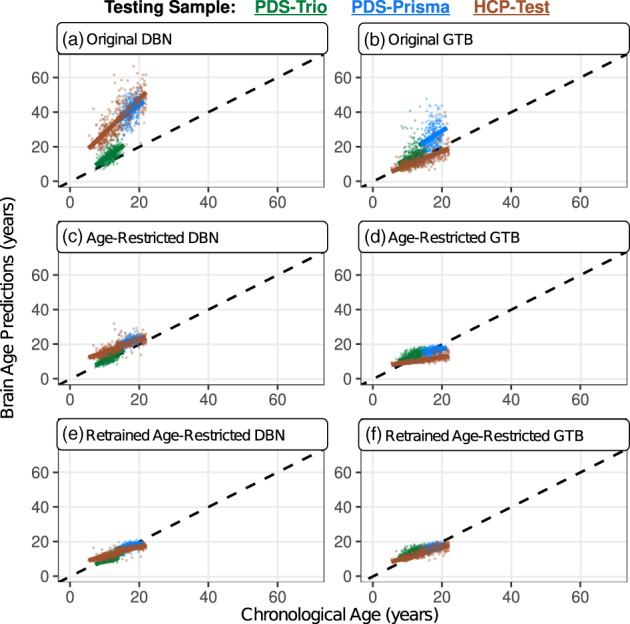
Brain age predictions were consistently overestimated, especially for scans acquired from Siemens Prisma scanners. This can be observed by how different the colored regression lines are relative to the black dashed line, which represents a perfect model fit (coefficient = 1; y‐intercept = 0). These biases were most pronounced when applying the original model variations (a & b). Attempts to restrict the age range of the training sample (c & d) and subsequently retrain each model (e & f) had varying levels of improvements, but issues of prediction bias persisted (Table 2). The PDS‐trio sample is represented by green circles, the PDS‐Prisma by blue triangles, and the HCP‐test by brown squares

Altogether, we examined prediction bias (i.e., slopes), scaling effects (i.e., y‐intercepts), and goodness of fit (i.e., prediction errors) for six brain age models (three variants from each machine learning framework), which were applied to three early‐developing cohorts (Table 2). When evaluating accuracy within each test sample the original GTB model was the most accurate (Figure 1b), as it was the least susceptible to prediction biases, scaling effects, and contained the most moderately‐fit prediction errors. The age‐restricted (Figure 1d) and retrained GTB (Figure 1f) displayed similar levels of accuracy, while exhibiting relatively larger prediction biases, scaling‐effects, but smaller prediction errors. In contrast, the original DBN (Figure 1a) was the least accurate of all models considering it had the largest prediction biases, scaling effects, and predictions errors. The age‐restricted DBN (Figure 1c) exhibited a noticeable improvement across all accuracy metrics, especially when applied to the PDS‐Trio. However, the retrained DBN (Figure 1e) overcorrected many issues from the original DBN, resulting in persisting prediction biases, scaling‐effects, but the smallest prediction errors of all models. In addition to differences between models, performance varied considerably between early developing cohorts, whereby most models were least accurate when applied to scans acquired from Siemens Prisma scanners (PDS‐Prisma & HCP‐Test). This imprecision between test samples may stem from scanner‐related variance, which also contributed to differences across all image quality metrics (*p* < 0.03) that were computed by the MRQy software package (Sadri et al., 2020) (Figure S2).

**TABLE 2:** The GTB was least susceptible to biases and overestimations, whereas the rDBN had the optimal amount of variation

Models	Slopes			Y‐intercepts			Mean absolute errors			Correlations		
	PDS‐Trio	PDS‐Prisma	HCP‐Test	PDS‐Trio	PDS‐Prisma	HCP‐Test	PDS‐Trio	PDS‐Prisma	HCP‐Test	PDS‐Trio	PDS‐Prisma	HCP‐Test
A. DBN	1.43	1.55	1.95	−1.2	13.0	8.5	4.0 (30)	22.9 (26)	21.7 (47)	0.58	0.40	0.78
B. rDBN	1.05	0.60	0.70	0.4	11.3	8.6	1.7 (11)	4.4 (13)	4.7 (26)	0.72	0.48	0.79
C. tDBN	0.80	0.43	0.53	1.4	10.1	6.5	1.4 (9)	1.2 (11)	1.8 (19)	0.68	0.47	0.84
D. GTB	0.95	1.07	0.80	3.4	7.3	1.5	3.4 (35)	8.8 (38)	2.8 (30)	0.33	0.29	0.70
E. rGTB	0.74	0.37	0.31	4.7	9.9	6.6	2.1 (9)	1.6 (9)	3.4 (16)	0.57	0.39	0.73
F. tGTB	0.73	0.38	0.56	5.2	10.2	5.5	2.3 (10)	1.4 (9)	1.9 (16)	0.57	0.43	0.83

*Note*: Accuracy metrics are reported for all six brain age models, which were applied to three test samples. A slope of one and y‐intercept of zero would indicate that brain age predictions were not systematically over or underestimated. A model's goodness of fit improves as MAEs approach zero and correlations approach one. However, moderately‐fit models (MAE: 3–6 years) may be most useful for detecting individual differences. Correlations become weaker when the age range of a test sample is restricted, which may explain why the HCP‐Test consistently yielded the strongest correlations relative to the PDS subsamples. The range of prediction errors are reported in parentheses next to the MAEs.

Nearly all six models exhibited prediction bias when applied to each of the three early developing cohorts (Table S1). This is a well‐known limitation of the brain age framework (Jonsson et al., 2019) and it is common to account for such artifacts using post‐hoc corrections. We followed this practice by linearly regressing out chronological age from the brain age gaps. As such, the modified brain age gaps were residualized with respect to age, which completely removed the previously reported issues of prediction bias and scaling effects. As expected, these modified brain age gaps also displayed much smaller prediction errors (median MAE: 0.88), though recent research has characterized such improvements as artificial (Butler et al., 2021) (Table S2).

### 3.2 Model reliability: How consistent are brain age predictions across model variants?

**FIGURE 2:**
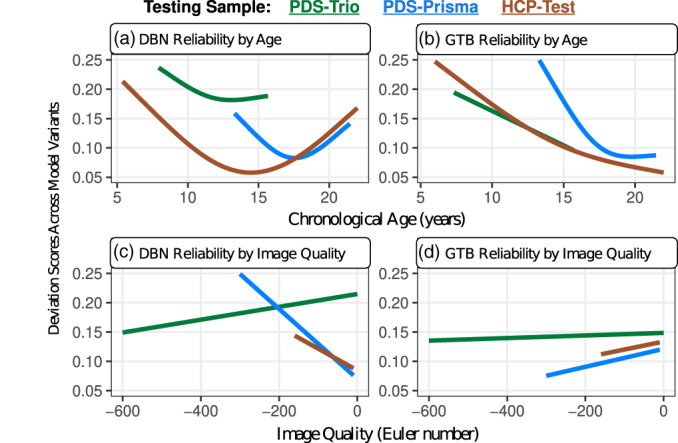
Patterns of reliability exhibited age‐related and image‐quality differences within each test sample, which were unique for the DBN and GTB. (a) The variants of the DBN were most inconsistent among the youngest and oldest individuals in each test sample. (b) In contrast, the GTB variants were only the most inconsistent among the youngest individuals in each sample. (c) The reliability of the DBN predictions were significantly associated with image quality, which might be a down‐stream consequence of using a minimal preprocessing pipeline. (d) The GTB variants yielded stable predictions that did not vary with image quality

Model reliability was assessed by examining the amount of variation across all three variants of the same machine learning framework, which produced mixed results. In particular, the deviation scores of the GTB yielded smaller deviations across participants from the PDS‐Trio sample, but the DBN yielded smaller deviations for the PDS‐Prisma and HCP‐Test samples (Table S3). Generalized additive models were used to further understand whether deviations in normalized brain age gaps varied as a function of age, sex, or Euler number (a proxy of image quality; Somerville et al., 2018). Reliability of the DBN and GTB were both robustly associated with age (Table S4), but these relationships were relatively more non‐linear for the DBN (Figure 2a). The DBN was least reliable when scans were at the edges of the age range (youngest and oldest), whereas reliability of the GTB linearly improved with age (Figure 2b). Reliability of the DBN was also associated with Euler Number (Figure 2c), suggesting that DBN predictions were more inconsistent when applied to lower quality MRI scans acquired from Siemens Prisma scanners. The reliability of the GTB variants was not related to image quality (Figure 2d).

Supplemental analyses were conducted to understand how these reliability results would change when deviation scores were based on the corrected brain age gaps. The raw and corrected brain age predictions were moderately to strongly correlated (Table S5), though deviation scores from the corrected brain age gaps were much smaller due to the reductions in prediction errors following the post‐hoc adjustment. Nonetheless, the DBN continued to be more reliable across participants from the PDS‐Trio and the GTB was more reliable across the HCP‐Test sample (Table S3). These new sets of deviation scores also revealed even stronger relationships between model reliability and image quality (Table S6). However, the age‐related differences in model reliability did not persist among deviation scores that were derived from age‐corrected brain age gaps (Table S6).

### 3.3 Model utility: To what extent can brain age gaps detect differences in cognition?

**FIGURE 3:**
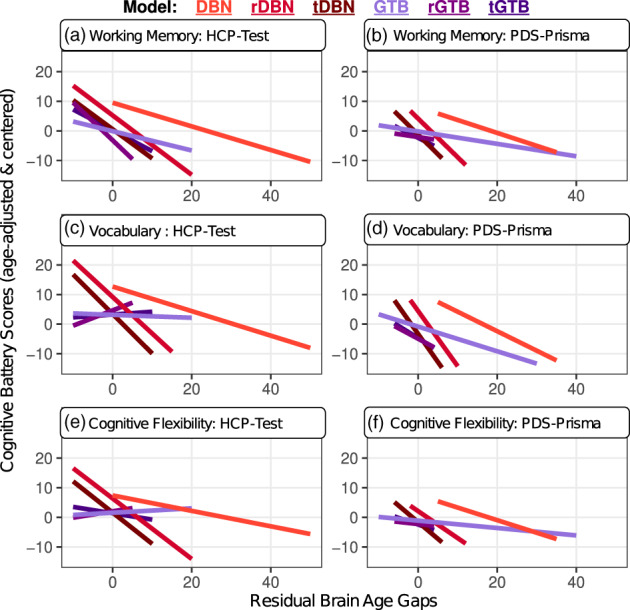
The brain age gaps derived by the DBN variants were most useful for detecting differences in cognitive functioning. These effects were replicated across multiple cognitive domains using two distinct test samples. The original DBN model yielded the most useful brain age gaps, which were associated with each of the cognitive domains displayed above. All significant effects indicated that underestimated brain age predictions were associated with better cognitive abilities

Significant associations between brain age gaps and cognitive functioning have been reported in a prior study, which found the largest effects with working memory and processing speed (Erus et al., 2015). The current study attempted to replicate these relationships by examining five cognitive domains (Figure S3) using linear and mixed‐effects models that controlled for chronological age, sex, and image quality (Somerville et al., 2018). The original and age‐restricted DBN models were the most able to detect significant associations between brain age gaps and cognitive performance (Table S7). Of the five domains measured separately for the PDS‐Prisma and HCP‐Test samples (10 possible correlations), the DBN had seven significant relationships and the rDBN had five; all of which suggested that underestimated brain ages were correlated with better performance (Figure 3). All other models had no more than one significant relationship, which were also negatively correlated. Of the 15 significant relationships, four were detected with working memory, six with language and three with cognitive flexibility, suggesting that the models were most sensitive to individual differences in these three domains.

## 4 DISCUSSION

In response to the widespread adoption and dissemination of brain age models, the current study benchmarked the generalizability of two models that were generated with the largest and most diverse training samples, which spanned from early childhood to late adulthood. We found that no single model outperformed across all facets of generalizability or all early developing test cohorts. Such findings present numerous trade‐offs that can be used as a guide to maximize the utility of these two models. As detailed below, the GTB predictions were relatively more accurate overall and yielded more reliable predictions when applied to lower quality scans. In contrast, the brain age gaps from the DBN had the most utility for detecting differences in cognitive functioning.

Our accuracy results were generally not as optimistic as previous brain age research (Chen et al., 2020). Specifically, we observed much weaker correlations between chronological and predicted brain ages (0.29–0.84), relative to the original cross‐validations that were performed across the lifespan (DBN; Bashyam et al., 2020: 0.98; GTB; Kaufmann et al., 2019: 0.94). Furthermore, only the retrained DBN and GTB yielded prediction errors that were analogous to prior neurodevelopmental studies (Brown et al., 2012; Erus et al., 2015; Niu et al., 2019) (MAEs from testing samples: 1–2). Although the goodness of fit improved when making the training and testing samples more similar, the prediction biases and scaling effects persisted for the DBN variants and worsened for the GTB variants. Broadly speaking, the original GTB was the most accurate, as it had the lowest prediction biases with moderately‐fit errors. However, all evaluated models could have been more precise, considering that each of the accuracy metrics varied considerably between test samples (Table 2). This challenge may be attributable to prediction bias and acquisition protocol differences between the train and test samples. Such confounds were not completely mitigated by any of the models in this study, emphasizing the need for better data aggregation and harmonization methods to achieve more generalizable models. This is in accordance with a previous study that applied ComBat harmonization (Fortin et al., 2017) and obtained more consistent brain age predictions across multi‐scanner data (Truelove‐Hill et al., 2020).

Model reliability was defined as the amount of variation across models of the same machine learning framework, which produced mixed results that varied with scanner type. The DBN was most reliable when applied to test samples acquired from Siemens Prisma scanners (PDS‐Prisma and HCP‐Test), but the GTB was significantly more reliable when applied to the Siemens Trio scans (PDS‐Trio). Patterns of reliability also exhibited robust age‐related and image‐quality differences within each test sample, which were unique for the DBN and GTB. The minimal preprocessing used by the DBN framework might contribute to it being more unreliable among lower quality scans, which was not exhibited by the GTB. Therefore, employing robust pipelines that extract neuroimaging features with higher signal‐to‐noise ratios (Magnotta et al., 2006) may lead to more consistent results with less dependence on subtle differences in image quality. However, implementing such methods in a clinical setting might come with practical challenges as they require processing time, computation resources, and programming expertise. The downstream implications of preprocessing and methodological choices on brain age predictions should be deliberated when building future models.

Model utility was assessed by how correlated brain age gaps were with cognitive functioning across five domains. The original and age‐restricted DBN were the most able to detect individual differences in cognitive functioning, whereas the GTB variants only detected a few associations. These relationships with cognition were found using age‐adjusted t‐scores, indicating that the significant effects were not driven by prediction bias (Figure S3). Yet, it was unexpected that the brain age gaps from the original DBN were the most sensitive to cognition, because this model yielded the most overestimated predictions and contained the largest prediction errors (MAE: 22.86). To date, only one study preformed an exploratory analysis of the relationship between model accuracy and utility (Bashyam et al., 2020), yielding the conclusion that moderately‐fit models (MAE: 5.92) are relatively better at detecting differences between healthy controls and patients with Alzheimer's disease. The current study aligns with these previously reported results by showing that the most “accurate” model tends to not be the most sensitive, at least in the context of cognitive functioning. As such, further work is needed to determine what features of the training sets or attributes of the brain age models are most critical for determining a model's utility to detect relationships with psychological or cognitive outcomes.

We observed notable differences in our results when analyzing the corrected brain age predictions. Post‐hoc corrections to remove prediction biases resulted in improved accuracy as the predictions errors were reduced such that most models became very tightly‐fit (median MAE: 0.88). However, recent research suggests that these improvements may be artificial (Butler et al., 2021). There are also additional challenges when interpreting the corrected brain age predictions in terms of identifying which brain regions significantly contributed to a given predictive pattern. Understanding the specific features involved in the brain age predictions could improve our knowledge of the underlying brain maturation process while also making the automated system transparent to human verification. However, the link between brain features and age predictions becomes complicated once post‐hoc corrections are applied. In addition, the best way to remove prediction bias is not clear (Smith et al., 2019). Lastly, applying group‐level calibrations to brain age gaps might not be feasible in clinical settings where assessments are made on an‐individual basis. Given these challenges, it is essential to develop and validate brain age models that are less susceptible to prediction bias, thereby alleviating the need for post‐hoc corrections altogether.

The current study contained several limitations. First, the age range of our test samples was at the lower limit of those used to train the DBN and GTB, which encompassed the entire lifespan. It is possible that different results might be obtained when evaluating these models with middle‐age or late adulthood cohorts (Amoroso et al., 2019). Second, our accuracy results might have been worse than prior research, because most studies used cross‐validations to evaluate a model's predictive power, but such methods are more optimistic compared to hold‐out tests (Poldrack et al., 2020). Furthermore, it is challenging for us to interpret how our accuracy results compare to those from prior studies, because model accuracy depends on multiple factors, including age range, age distribution, sample size, specific accuracy metrics, and bias correction methods (de Lange et al., 2022; Smith et al., 2019). Third, the current study evaluated two machine learning frameworks that differed in their preprocessing methods, neuroimaging features, predictive algorithms, test samples, and retraining procedures. This diversity led to a more encompassing evaluation of the brain age framework, but it also presented challenges in narrowing down how each of these differences uniquely influenced brain age predictions. Subsequent studies may benefit from using ablation study designs, whereby comparisons are made between models with more similarities than differences.

The brain age framework has the potential to provide useful individual‐level indices of brain development as long as its predictions are generalizable across diverse populations from all developmental stages. This study delineated numerous opportunities for improvement in the generalizability of brain‐age models. The evaluated models have many practical uses provided that the biases revealed here can be accounted for (e.g., adjusted for systematic offsets in predicting age). Overall, the age‐restricted DBN had reasonable accuracy and was the second most useful at detecting individual differences in cognition. The original GTB was the most accurate and its predictions were less susceptible to inconsistencies when applied to lower quality scans, but it was not as sensitive to differences in cognition. To conclude, the largest limitations affecting the generalizability of brain age models were acquisition protocol differences and prediction biases. If such confounds could eventually be removed without post‐hoc corrections, brain age predictions may have greater utility as personalized biomarkers of healthy aging.

## CONFLICT OF INTEREST

All authors report no biomedical financial interests or potential conflict of interest.

## Supporting information


**Appendix S1** Supporting Information

## Data Availability

The data incorporated in this work were gathered from various resources. The Human Connectome Project in Development (HCP‐D) is publicly available through the NIMH Data Archive, which is documented here: https://www.humanconnectome.org/study/hcp‐lifespan‐development/article/lifes. Preschool Depression Sample (PDS) is not publicly available, but requests can be directed to the corresponding author from this cited article (Liang et al., 2019). All code and model variants pertaining to this study have been made available through the following GitHub repository: https://github.com/ccplabwustl/RobertJirsaraie/tree/master/proj20‐BrainAgeEval.

## References

[hbm26144-bib-0001] Amoroso, N. , la Rocca, M. , Bellantuono, L. , Diacono, D. , Fanizzi, A. , Lella, E. , Lombardi, A. , Maggipinto, T. , Monaco, A. , Tangaro, S. , & Bellotti, R. (2019). Deep learning and multiplex networks for accurate modeling of brain age. Frontiers in Aging Neuroscience, 11, 1–12. 31178715 10.3389/fnagi.2019.00115 PMC6538815

[hbm26144-bib-0002] Bashyam, V. M. , Erus, G. , Doshi, J. , Habes, M. , Nasrallah, I. M. , Truelove‐Hill, M. , Srinivasan, D. , Mamourian, L. , Pomponio, R. , Fan, Y. , Launer, L. J. , Masters, C. L. , Maruff, P. , Zhuo, C. , Völzke, H. , Johnson, S. C. , Fripp, J. , Koutsouleris, N. , Satterthwaite, T. D. , … Davatzikos, C. (2020). MRI signatures of brain age and disease over the lifespan based on a deep brain network and 14 468 individuals worldwide. Brain, 143, 2312–2324. 32591831 10.1093/brain/awaa160 PMC7364766

[hbm26144-bib-0003] Brown, T. T. , Kuperman, J. M. , Chung, Y. , Erhart, M. , McCabe, C. , Hagler, D. J., Jr. , Venkatraman, V. K. , Akshoomoff, N. , Amaral, D. G. , Bloss, C. S. , Casey, B. J. , Chang, L. , Ernst, T. M. , Frazier, J. A. , Gruen, J. R. , Kaufmann, W. E. , Kenet, T. , Kennedy, D. N. , Murray, S. S. , … Dale, A. M. (2012). Neuroanatomical assessment of biological maturity. Current Biology, 22, 1693–1698. 22902750 10.1016/j.cub.2012.07.002 PMC3461087

[hbm26144-bib-0004] Butler, E. R. , Chen, A. , Ramadan, R. , le, T. T. , Ruparel, K. , Moore, T. M. , Satterthwaite, T. D. , Zhang, F. , Shou, H. , Gur, R. C. , Nichols, T. E. , & Shinohara, R. T. (2021). Pitfalls in brain age analyses. Human Brain Mapping, 42, 4092–4101. 34190372 10.1002/hbm.25533 PMC8357007

[hbm26144-bib-0005] Casaletto, K. B. , Umlauf, A. , Beaumont, J. , Gershon, R. , Slotkin, J. , Akshoomoff, N. , & Heaton, R. K. (2015). Demographically corrected normative standards for the English version of the NIH toolbox cognition battery. Journal of the International Neuropsychological Society, 21, 378–391. 26030001 10.1017/S1355617715000351 PMC4490030

[hbm26144-bib-0006] Chen, C.‐L. , Hsu, Y. C. , Yang, L. Y. , Tung, Y. H. , Luo, W. B. , Liu, C. M. , Hwang, T. J. , Hwu, H. G. , & Isaac Tseng, W. Y. (2020). Generalization of diffusion magnetic resonance imaging–based brain age prediction model through transfer learning. NeuroImage, 217, 116831. 32438048 10.1016/j.neuroimage.2020.116831

[hbm26144-bib-0007] Cole, J. H. , & Franke, K. (2017). Predicting age using neuroimaging: Innovative brain ageing biomarkers. Trends in Neurosciences, 40, 681–690. 29074032 10.1016/j.tins.2017.10.001

[hbm26144-bib-0008] Dale, A. M. , Fischl, B. , & Sereno, M. I. (1999). Cortical surface‐based analysis: I. Segmentation and surface reconstruction. Neuroimage, 9, 179–194. 9931268 10.1006/nimg.1998.0395

[hbm26144-bib-0009] de Lange, A.‐M. G. , Anatürk, M. , Rokicki, J. , Han, L. K. M. , Franke, K. , Alnaes, D. , Ebmeier, K. P. , Draganski, B. , Kaufmann, T. , Westlye, L. T. , Hahn, T. , & Cole, J. H. (2022). Mind the gap: Performance metric evaluation in brain‐age prediction. Human Brain Mapping, 43, 3113–3129. 10.1002/hbm.25837 35312210 PMC9188975

[hbm26144-bib-0010] Doshi, J. , Erus, G. , Ou, Y. , Gaonkar, B. , & Davatzikos, C. (2013). Multi‐atlas Skull‐stripping. Academic Radiology, 20, 1566–1576. 24200484 10.1016/j.acra.2013.09.010 PMC3880117

[hbm26144-bib-0011] Erus, G. , Battapady, H. , Satterthwaite, T. D. , Hakonarson, H. , Gur, R. E. , Davatzikos, C. , & Gur, R. C. (2015). Imaging patterns of brain development and their relationship to cognition. Cerebral Cortex, 25, 1676–1684. 24421175 10.1093/cercor/bht425 PMC4428302

[hbm26144-bib-0012] Fischl, B. (2012). FreeSurfer. Neuroimage, 62, 774–781. 22248573 10.1016/j.neuroimage.2012.01.021 PMC3685476

[hbm26144-bib-0013] Fonov, V. , Evans, A. C. , Botteron, K. , Almli, C. R. , McKinstry, R. , Collins, D. L. , & Brain Development Cooperative Group . (2011). Unbiased average age‐appropriate atlases for pediatric studies. NeuroImage, 54, 313–327. 20656036 10.1016/j.neuroimage.2010.07.033 PMC2962759

[hbm26144-bib-0014] Fortin, J. P. , Parker, D. , Tunç, B. , Watanabe, T. , Elliott, M. A. , Ruparel, K. , Roalf, D. R. , Satterthwaite, T. D. , Gur, R. C. , Gur, R. E. , Schultz, R. T. , Verma, R. , & Shinohara, R. T. (2017). Harmonization of multi‐site diffusion tensor imaging data. NeuroImage, 161, 149–170. 28826946 10.1016/j.neuroimage.2017.08.047 PMC5736019

[hbm26144-bib-0015] Franke, K. , & Gaser, C. (2012). Longitudinal changes in individual BrainAGE in healthy aging, mild cognitive impairment, and Alzheimer's disease. GeroPsych, 25(235–245), 235–245.

[hbm26144-bib-0016] Gaser, C. , Franke, K. , Klöppel, S. , Koutsouleris, N. , Sauer, H. , & Alzheimer's Disease Neuroimaging Initiative . (2013). BrainAGE in mild cognitive impaired patients: Predicting the conversion to Alzheimer's disease. PLoS One, 8, e67346. 23826273 10.1371/journal.pone.0067346 PMC3695013

[hbm26144-bib-0017] Glasser, M. F. , Coalson, T. S. , Robinson, E. C. , Hacker, C. D. , Harwell, J. , Yacoub, E. , Ugurbil, K. , Andersson, J. , Beckmann, C. F. , Jenkinson, M. , Smith, S. M. , & van Essen, D. C. (2016). A multi‐modal parcellation of human cerebral cortex. Nature, 536, 171–178. 27437579 10.1038/nature18933 PMC4990127

[hbm26144-bib-0018] Han, X. , Jovicich, J. , Salat, D. , van der Kouwe, A. , Quinn, B. , Czanner, S. , Busa, E. , Pacheco, J. , Albert, M. , Killiany, R. , Maguire, P. , Rosas, D. , Makris, N. , Dale, A. , Dickerson, B. , & Fischl, B. (2006). Reliability of MRI‐derived measurements of human cerebral cortical thickness: The effects of field strength, scanner upgrade and manufacturer. NeuroImage, 32, 180–194. 16651008 10.1016/j.neuroimage.2006.02.051

[hbm26144-bib-0019] Harms, M. P. , Somerville, L. H. , Ances, B. M. , Andersson, J. , Barch, D. M. , Bastiani, M. , Bookheimer, S. Y. , Brown, T. B. , Buckner, R. L. , Burgess, G. C. , Coalson, T. S. , Chappell, M. A. , Dapretto, M. , Douaud, G. , Fischl, B. , Glasser, M. F. , Greve, D. N. , Hodge, C. , Jamison, K. W. , … Yacoub, E. (2018). Extending the human connectome project across ages: Imaging protocols for the lifespan development and aging projects. NeuroImage, 183, 972–984. 30261308 10.1016/j.neuroimage.2018.09.060 PMC6484842

[hbm26144-bib-0020] Høgestøl, E. A. , Kaufmann, T. , Nygaard, G. O. , Beyer, M. K. , Sowa, P. , Nordvik, J. E. , Kolskår, K. , Richard, G. , Andreassen, O. A. , Harbo, H. F. , & Westlye, L. T. (2019). Cross‐sectional and longitudinal MRI brain scans reveal accelerated brain aging in multiple sclerosis. Frontiers in Neurology, 10, 450. 31114541 10.3389/fneur.2019.00450 PMC6503038

[hbm26144-bib-0021] Jenkinson, M. , Bannister, P. , Brady, M. , & Smith, S. (2002). Improved optimization for the robust and accurate linear registration and motion correction of brain images. NeuroImage, 17, 825–841. 12377157 10.1016/s1053-8119(02)91132-8

[hbm26144-bib-0022] Jernigan, T. L. , Brown, T. T. , Hagler, D. J., Jr. , Akshoomoff, N. , Bartsch, H. , Newman, E. , Thompson, W. K. , Bloss, C. S. , Murray, S. S. , Schork, N. , Kennedy, D. N. , Kuperman, J. M. , McCabe, C. , Chung, Y. , Libiger, O. , Maddox, M. , Casey, B. J. , Chang, L. , Ernst, T. M. , … Pediatric Imaging, Neurocognition and Genetics Study . (2016). The pediatric imaging, Neurocognition, and genetics (PING) data repository. NeuroImage, 124, 1149–1154. 25937488 10.1016/j.neuroimage.2015.04.057 PMC4628902

[hbm26144-bib-0023] Jonsson, B. A. , Bjornsdottir, G. , Thorgeirsson, T. E. , Ellingsen, L. M. , Walters, G. B. , Gudbjartsson, D. F. , Stefansson, H. , Stefansson, K. , & Ulfarsson, M. O. (2019). Brain age prediction using deep learning uncovers associated sequence variants. Nature Communications, 10, 5409. 10.1038/s41467-019-13163-9 PMC6881321 31776335

[hbm26144-bib-0024] Jovicich, J. , Czanner, S. , Han, X. , Salat, D. , van der Kouwe, A. , Quinn, B. , Pacheco, J. , Albert, M. , Killiany, R. , Blacker, D. , Maguire, P. , Rosas, D. , Makris, N. , Gollub, R. , Dale, A. , Dickerson, B. C. , & Fischl, B. (2009). MRI‐derived measurements of human subcortical, ventricular and intracranial brain volumes: Reliability effects of scan sessions, acquisition sequences, data analyses, scanner upgrade, scanner vendors and field strengths. NeuroImage, 46, 177–192. 19233293 10.1016/j.neuroimage.2009.02.010 PMC2866077

[hbm26144-bib-0025] Kaufmann, T. , van der Meer, D. , Doan, N. T. , Schwarz, E. , Lund, M. J. , Agartz, I. , Alnæs, D. , Barch, D. M. , Baur‐Streubel, R. , Bertolino, A. , Bettella, F. , Beyer, M. K. , Bøen, E. , Borgwardt, S. , Brandt, C. L. , Buitelaar, J. , Celius, E. G. , Cervenka, S. , Conzelmann, A. , … Westlye, L. T. (2019). Common brain disorders are associated with heritable patterns of apparent aging of the brain. Nature Neuroscience, 22, 1617–1623. 31551603 10.1038/s41593-019-0471-7 PMC6823048

[hbm26144-bib-0026] Kuo, C.‐Y. , Tai, T.‐M. , Lee, P.‐L. , Tseng, C.‐W. , Chen, C.‐Y. , Chen, L.‐K. , Lee, C.‐K. , Chou, K.‐H. , See, S. , & Lin, C.‐P. (2021). Improving individual brain age prediction using an ensemble deep learning framework. Frontiers in Psychiatry, 12, 308. 10.3389/fpsyt.2021.626677 PMC8021919 33833699

[hbm26144-bib-0027] Leonardsen, E. H. , Peng, H. , Kaufmann, T. , Agartz, I. , Andreassen, O. A. , Celius, E. G. , Espeseth, T. , Harbo, H. F. , Høgestøl, E. A. , Lange, A. M. , Marquand, A. F. , Vidal‐Piñeiro, D. , Roe, J. M. , Selbæk, G. , Sørensen, Ø. , Smith, S. M. , Westlye, L. T. , Wolfers, T. , & Wang, Y. (2022). Deep neural networks learn general and clinically relevant representations of the ageing brain. NeuroImage, 256, 119210. 35462035 10.1101/2021.10.29.21265645 PMC7614754

[hbm26144-bib-0028] Liang, H. , Zhang, F. , & Niu, X. (2019). Investigating systematic bias in brain age estimation with application to post‐traumatic stress disorders. Human Brain Mapping, 40, 3143–3152. 30924225 10.1002/hbm.24588 PMC6865701

[hbm26144-bib-0029] Liem, F. , Varoquaux, G. , Kynast, J. , Beyer, F. , Kharabian Masouleh, S. , Huntenburg, J. M. , Lampe, L. , Rahim, M. , Abraham, A. , Craddock, R. C. , Riedel‐Heller, S. , Luck, T. , Loeffler, M. , Schroeter, M. L. , Witte, A. V. , Villringer, A. , & Margulies, D. S. (2017). Predicting brain‐age from multimodal imaging data captures cognitive impairment. NeuroImage, 148, 179–188. 27890805 10.1016/j.neuroimage.2016.11.005

[hbm26144-bib-0030] Luby, J. L. , Xuemei, S. , Belden, A. C. , Tandon, M. , & Spitznagel, E. (2010). Preschool depression: The importance of identification of depression early in development. Current Directions in Psychological Science, 19, 91–95. 21969769 10.1177/0963721410364493 PMC3184303

[hbm26144-bib-0031] Magnotta, V. A. , Friedman, L. , & Birn, F. (2006). Measurement of signal‐to‐noise and contrast‐to‐noise in the fBIRN multicenter imaging study. Journal of Digital Imaging, 19, 140–147. 16598643 10.1007/s10278-006-0264-x PMC3045184

[hbm26144-bib-0032] Niu, X. , Zhang, F. , Kounios, J. , & Liang, H. (2019). Improved prediction of brain age using multimodal neuroimaging data. Human Brain Mapping, 41, 1626–1643. 31837193 10.1002/hbm.24899 PMC7267976

[hbm26144-bib-0033] Poldrack, R. A. , Huckins, G. , & Varoquaux, G. (2020). Establishment of best practices for evidence for prediction: A review. JAMA Psychiatry, 77, 534–540. 10.1001/jamapsychiatry.2019.3671 31774490 PMC7250718

[hbm26144-bib-0034] Richard, G. , Kolskår, K. , Ulrichsen, K. M. , Kaufmann, T. , Alnæs, D. , Sanders, A. M. , Dørum, E. S. , Monereo Sánchez, J. , Petersen, A. , Ihle‐Hansen, H. , Nordvik, J. E. , & Westlye, L. T. (2020). Brain age prediction in stroke patients: Highly reliable but limited sensitivity to cognitive performance and response to cognitive training. NeuroImage: Clinical, 25, 102159. 31927499 10.1016/j.nicl.2019.102159 PMC6953960

[hbm26144-bib-0035] Rosen, A. F. G. , Roalf, D. R. , Ruparel, K. , Blake, J. , Seelaus, K. , Villa, L. P. , Ciric, R. , Cook, P. A. , Davatzikos, C. , Elliott, M. A. , Garcia de la Garza, A. , Gennatas, E. D. , Quarmley, M. , Schmitt, J. E. , Shinohara, R. T. , Tisdall, M. D. , Craddock, R. C. , Gur, R. E. , Gur, R. C. , & Satterthwaite, T. D. (2018). Quantitative assessment of structural image quality. NeuroImage, 169, 407–418. 29278774 10.1016/j.neuroimage.2017.12.059 PMC5856621

[hbm26144-bib-0036] Sadri, A. R. , Janowczyk, A. , Zhou, R. , Verma, R. , Beig, N. , Antunes, J. , Madabhushi, A. , Tiwari, P. , & Viswanath, S. E. (2020). Technical note: MRQy — An open‐source tool for quality control of MR imaging data. Medical Physics, 47, 6029–6038. 33176026 10.1002/mp.14593 PMC8176950

[hbm26144-bib-0037] Satterthwaite, T. D. , Elliott, M. A. , Ruparel, K. , Loughead, J. , Prabhakaran, K. , Calkins, M. E. , Hopson, R. , Jackson, C. , Keefe, J. , Riley, M. , Mentch, F. D. , Sleiman, P. , Verma, R. , Davatzikos, C. , Hakonarson, H. , Gur, R. C. , & Gur, R. E. (2014). Neuroimaging of the Philadelphia neurodevelopmental cohort. NeuroImage, 86, 544–553. 23921101 10.1016/j.neuroimage.2013.07.064 PMC3947233

[hbm26144-bib-0038] Schulz, M.‐A. , Yeo, B. T. T. , Vogelstein, J. T. , Mourao‐Miranada, J. , Kather, J. N. , Kording, K. , Richards, B. , & Bzdok, D. (2020). Different scaling of linear models and deep learning in UKBiobank brain images versus machine‐learning datasets. Nature Communications, 11, 4238. 10.1038/s41467-020-18037-z PMC7447816 32843633

[hbm26144-bib-0039] Smith, S. M. , Vidaurre, D. , Alfaro‐Almagro, F. , Nichols, T. E. , & Miller, K. L. (2019). Estimation of brain age delta from brain imaging. NeuroImage, 200, 528–539. 31201988 10.1016/j.neuroimage.2019.06.017 PMC6711452

[hbm26144-bib-0040] Somerville, L. H. , Bookheimer, S. Y. , Buckner, R. L. , Burgess, G. C. , Curtiss, S. W. , Dapretto, M. , Elam, J. S. , Gaffrey, M. S. , Harms, M. P. , Hodge, C. , Kandala, S. , Kastman, E. K. , Nichols, T. E. , Schlaggar, B. L. , Smith, S. M. , Thomas, K. M. , Yacoub, E. , van Essen, D. C. , & Barch, D. M. (2018). The lifespan human connectome project in development: A large‐scale study of brain connectivity development in 5–21 year olds. NeuroImage, 183, 456–468. 30142446 10.1016/j.neuroimage.2018.08.050 PMC6416053

[hbm26144-bib-0041] Team R. C . (2013). R: A language and environment for statistical computing. R Foundation for Statistical Computing, Vienna, Austria.

[hbm26144-bib-0042] Truelove‐Hill, M. , Erus, G. , Bashyam, V. , Varol, E. , Sako, C. , Gur, R. C. , Gur, R. E. , Koutsouleris, N. , Zhuo, C. , Fan, Y. , Wolf, D. H. , Satterthwaite, T. D. , & Davatzikos, C. (2020). A multidimensional neural maturation index reveals reproducible developmental patterns in children and adolescents. The Journal of Neuroscience, 40, 1265–1275. 31896669 10.1523/JNEUROSCI.2092-19.2019 PMC7002145

[hbm26144-bib-0043] Varoquaux, G. , Raamana, P. R. , Engemann, D. A. , Hoyos‐Idrobo, A. , Schwartz, Y. , & Thirion, B. (2017). Assessing and tuning brain decoders: Cross‐validation, caveats, and guidelines. NeuroImage, 145, 166–179. 27989847 10.1016/j.neuroimage.2016.10.038

[hbm26144-bib-0044] Weintraub, S. , Dikmen, S. S. , Heaton, R. K. , Tulsky, D. S. , Zelazo, P. D. , Bauer, P. J. , Carlozzi, N. E. , Slotkin, J. , Blitz, D. , Wallner‐Allen, K. , Fox, N. A. , Beaumont, J. L. , Mungas, D. , Nowinski, C. J. , Richler, J. , Deocampo, J. A. , Anderson, J. E. , Manly, J. J. , Borosh, B. , … Gershon, R. C. (2013). Cognition assessment using the NIH toolbox. Neurology, 80, S54–S64. 23479546 10.1212/WNL.0b013e3182872ded PMC3662346

