# Supplementary material for: Benchmarking the generalizability of brain age models: Challenges posed by scanner variance and prediction bias
Source: Hum Brain Mapp. 2022 Nov 8;44(3):1118–28. doi: 10.1002/hbm.26144 (PMC9875922; doi:10.1002/hbm.26144)
Supplement: Supplementary file 1 — Appendix S1 Supporting Information [file HBM-44-1118-s001.docx]

**Supplemental Material**

**Table S1.** Most brain age models were affected by linear age-related bias.

| **Models** | **Age Dependent Artifacts** | | | **Non-linearity Tests** | | |
| --- | --- | --- | --- | --- | --- | --- |
|  | PDS-Trio | PDS-Prisma | HCP-Test | PDS-Trio | PDS-Prisma | HCP-Test |
| A. DBN | 0.20*** | 0.14* | 0.52*** | 0.01 | 0 | 0.54 |
| B. rDBN | 0.09* | -0.33*** | -0.47*** | 0.58 | 0.21 | 7.01** |
| C. tDBN | -0.16** | -0.56*** | -0.81*** | 11.79*** | 2.29* | 15.83*** |
| D. GTB | -0.01 | 0.06 | -0.24*** | 0 | 0 | 14.29*** |
| E. rGTB | -0.22*** | -0.56*** | -0.92*** | 0 | 0 | 18.69*** |
| F. tGTB | -0.27*** | -0.58*** | -0.76*** | 0 | 0 | 1.69~ |

*Note.* Standardized beta coefficients are reported to evaluate the presence of age-related biases, whereas the restricted likelihood ratio test statistics are reported to understand whether such relationships were non-linear. Brain age gaps from all models varied as a function of age, except when the original GTB was applied to the PDS-Trio and PDS-Prisma samples. These relationships were mostly negative, which is the typical pattern of age-related bias that is commonly found in prior studies. The retrained DBN was the only variant to have non-linear biases across all three test samples, whereas all other relationships were mostly linear. Asterisks were used to represent the uncorrected p-values: (p ≤ 0.10)˜ , (p ≤ 0.05)^*^, (p ≤ 0.01)^**^, (p ≤ 0.001)^***^.

| **Models** | **Modified Mean Absolute Errors** | | | **Modified Correlations** | | |
| --- | --- | --- | --- | --- | --- | --- |
|  | PDS-Trio | PDS-Prisma | HCP-Test | PDS-Trio | PDS-Prisma | HCP-Test |
| A. DBN | 0.92 | 1.35 | 4.83 | 0.73 | 0.67 | 0.54 |
| B. rDBN | 0.42 | 0.37 | 1.59 | 0.94 | 0.95 | 0.87 |
| C. tDBN | 0.43 | 0.31 | 1.05 | 0.94 | 0.97 | 0.95 |
| D. GTB | 1.82 | 1.92 | 2.23 | 0.48 | 0.52 | 0.77 |
| E. rGTB | 0.62 | 0.55 | 0.84 | 0.90 | 0.91 | 0.96 |
| F. tGTB | 0.56 | 0.52 | 1.20 | 0.92 | 0.92 | 0.94 |

**Table S2.** Prediction errors were substantially reduced when applying a post-hoc correction to remove age-related biases from brain age gaps.

*Note.* Accuracy metrics regarding the goodness of fit of brain age predictions from all six model variants. The improved metrics are the result of regressing chronological age from the brain age gaps for each variant.

**Table S3**. Reliability differences between the DBN and GTB models.

| Deviation Scores | DBN Variants | GTB Variants | Degrees of Freedom | t-statistic | p-values |
| --- | --- | --- | --- | --- | --- |
| PDS-Trio | 0.19 (0.05) | 0.14 (0.06) | 431 | 13.20 | >0.001 |
| PDS-Prisma | 0.09 (0.05) | 0.11 (0.07) | 279 | -3.84 | >0.001 |
| HCP-Test | 0.10 (0.05) | 0.13 (0.06) | 393 | -10.18 | >0.001 |
| Modified PDS-Trio | 0.22 (0.05) | 0.09 (0.05) | 431 | 40.74 | >0.001 |
| Modified PDS-Prisma | 0.10 (0.07) | 0.10 (0.06) | 279 | -0.64 | 0.51 |
| Modified HCP-Test | 0.06 (0.02) | 0.12 (0.05) | 393 | -21.16 | >0.001 |

*Note.* Pairwise t-tests were performed to compare model reliability of the GTB and DBN within each of the three test samples. Two sets of deviation scores were evaluated to understand how reliability changes before (row 1-3) and after (rows 4-6) modifying the brain age gaps to remove biases. Significant differences were detected between the reliability of the GTB and DBN in all but 1 of the 6 comparisons. The direction of these effects largely differed by the scanner manufacture of the test sample.

**Table S4**. Age-related differences in reliability were mostly non-linear for the DBN models and linear for the GTB models.

| **Predictors** | **DBN Models** | | | **GTB Models** | | |  |
| --- | --- | --- | --- | --- | --- | --- | --- |
|  | PDS-Trio | PDS-Prisma | HCP-Test | PDS-Trio | PDS-Prisma | HCP-Test | |
| (R^2^ of Non-linear Model) – (R^2^ of the Equivalent Linear Model) | | | | | | | |
| Spline (Age) | 0.03*** | 0.08*** | 0.53*** | 0*** | 0.04*** | 0.04*** | |
| Standardized Beta Weights of Linear Models | | | | | | | |
| Linear Age | -0.21*** | -0.05 | <0.01 | -0.26*** | -0.42*** | -0.78*** | |
| Euler Number | -0.30*** | 0.29*** | 0.11~ | 0.04 | 0.05 | 0.04 | |
| Sex | 0.12~ | -0.02 | 0.03 | 0.06 | 0.04 | 0.08* | |
| NAVs | - | - | 0.08 | - | - | -0.01 | |

*Note.* The effect sizes of the non-linear relationships reported above (row 1) were based on the adjusted R-squared of the generalized additive model minus the adjusted R-squared of the equivalent linear models. Non-linear relationships only had age as the single predictor of deviation scores. In contrast, linear relationships were analyzed using regressions and mixed models that include each predictor reported above (rows 2-5) along with their standardized regression coefficients as indicators of effect size. Asterisks were used to represent the uncorrected p-values: (p ≤ 0.10)˜ , (p ≤ 0.05)^*^, (p ≤ 0.01)^**^, (p ≤ 0.001)^***^.

**Table S5**. Raw and corrected brain age predictions were either moderately or strongly correlated.

| **Models** | **Correlations** | | |
| --- | --- | --- | --- |
|  | PDS-Trio  (age range: 8.3) | PDS-Prisma  (age range: 8.2) | HCP-Test  (age range: 16.6) |
| A. DBN | 0.79 | 0.72 | 0.95 |
| B. rDBN | 0.81 | 0.62 | 0.98 |
| C. tDBN | 0.78 | 0.60 | 0.97 |
| D. GTB | 0.88 | 0.77 | 0.99 |
| E. rGTB | 0.76 | 0.67 | 0.89 |
| F. tGTB | 0.73 | 0.69 | 0.97 |

*Note.* Pearson correlations are reported above to understand the similarity between raw and corrected brain age predictions. As discussed in the methods, the correlation largely varied with the age range of the test sample with the constricted ranges being less correlated. Nonetheless, it was reassuring that the original DBN had the highest correlation for each test sample relative to all other variants. This is because the original GTB was the least susceptible to age-related biases, indicating that the corrected brain ages should not be as different compared to the raw brain ages.

**Table S6**. Correcting for age-related biases attenuated the age-related differences in reliability but strengthened image-quality differences.

| **Predictors** | **DBN Models** | | | | **GTB Models** | | |  |
| --- | --- | --- | --- | --- | --- | --- | --- | --- |
|  | | PDS-Trio | PDS-Prisma | HCP-Test | PDS-Trio | PDS-Prisma | HCP-Test | |
| Chronological Age | | 0.09~ | 0.02 | 0.07 | 0.01 | -0.01 | 0.05 | |
| Euler Number | | 0.48*** | -0.14* | 0.19** | -0.08~ | -0.08 | 0.24*** | |
| Sex | | -0.03 | 0.01 | 0.04 | -0.02 | -0.11 | 0.24*** | |
| NAVs | | - | - | 0.08 | - | - | -0.07 | |

*Note.* Supplemental analyses were conducted that based deviation scores on the age-corrected brain age gaps following min/max normalization. In doing so, the previously observed age-related differences in model reliability did not persist, which was the case for all linear and non-linear relationships. However, consistency in the ordering of age-corrected brain age gaps was even more related to image quality, though the direction of these relationships changed for the PDS-Trio and HCP-Test, as observed by their standardized regression coefficients reported above. Asterisks were used to represent the uncorrected p-values: (p ≤ 0.10)˜ , (p ≤ 0.05)^*^, (p ≤ 0.01)^**^, (p ≤ 0.001)^***^.

**Table S7**. Brain age gaps from the DBN and rDBN variants were the most consistently related to cognitive functioning.

| **Model** | **Working Memory** | | **Processing Speed** | | **Episodic Memory** | | **Vocabulary** | | **Cognitive Flexibility** | | |
| --- | --- | --- | --- | --- | --- | --- | --- | --- | --- | --- | --- |
|  | PDS Prisma | HCP Test | PDS Prisma | HCP Test | PDS Prisma | HCP Test | PDS Prisma | HCP Test | | PDS Prisma | HCP Test |
| A. DBN | -0.15* | -0.28** | 0.02 | -0.16~ | -0.13~ | -0.18* | -0.21** | -0.27** | | -0.14* | -0.19* |
| B. rDBN | -0.15~ | -0.25** | -0.03 | -0.17* | -0.11 | -0.17 | -0.20* | -0.29** | | -0.12 | -0.27** |
| C. tDBN | -0.10 | -0.19~ | 0.03 | -0.04 | -0.03 | -0.01 | -0.14 | -0.22* | | -0.11 | -0.19~ |
| D. GTB | -0.08 | -0.12 | 0.04 | -0.05 | -0.07 | -0.03 | -0.15* | -0.02 | | -0.06 | 0.06 |
| E. rGTB | -0.01 | -0.17* | 0.02 | -0.12 | 0.03 | -0.09 | -0.06 | 0.04 | | -0.01 | 0.04 |
| F. tGTB | -0.05 | -0.15 | 0.06 | -0.06 | 0.02 | 0.02 | -0.06 | 0.01 | | -0.04 | -0.01 |

*Note.* Standardized regression coefficients for each cognitive domain are reported above. Asterisks were used to represent the uncorrected p-values: (p ≤ 0.10)˜ , (p ≤ 0.05)^*^, (p ≤ 0.01)^**^. All analyses included chronological age, sex, and Euler number as covariates and brain age predictions were always the response variable.


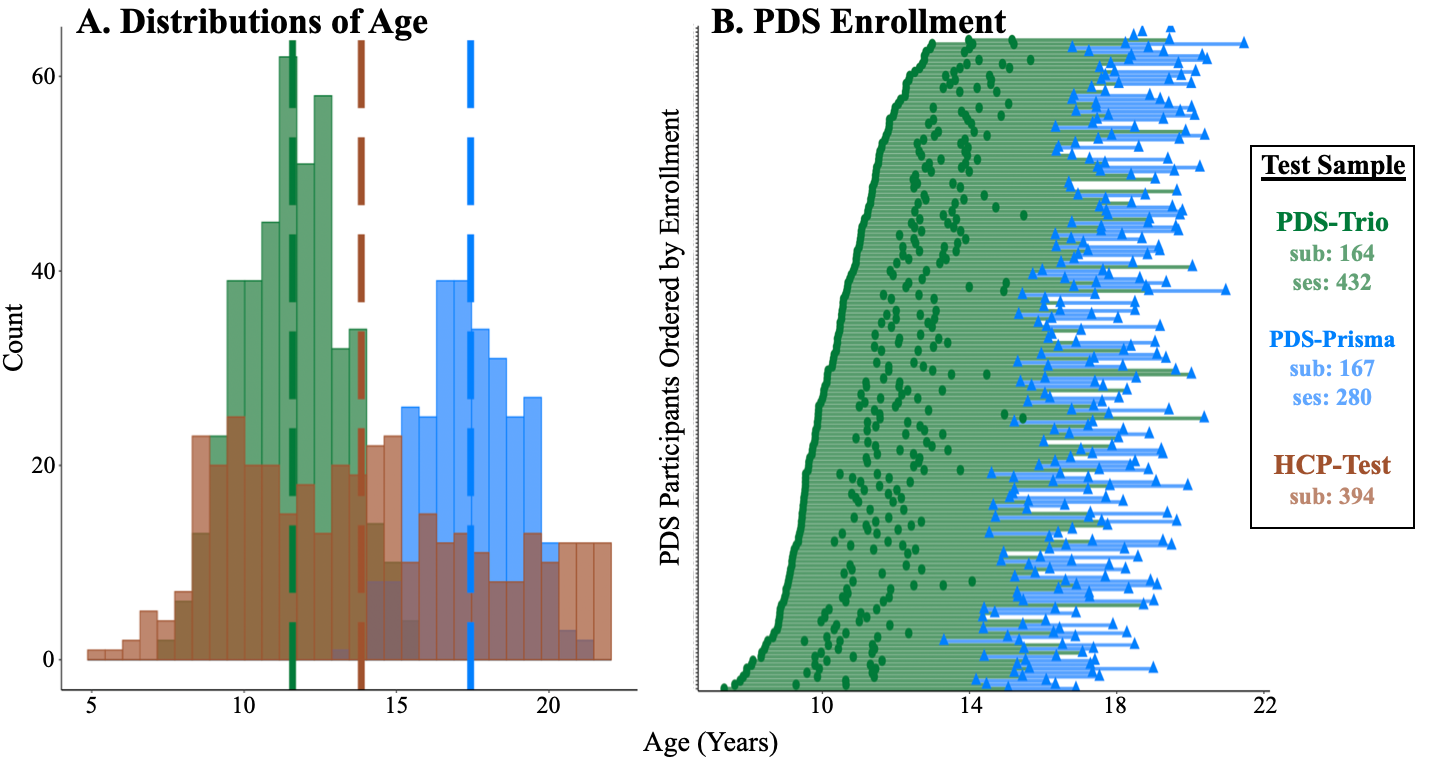


**Figure S1. All scans pertained to early development though the distribution of scan ages varied considerably between the test samples. A.** Heterogeneity in the age distributions of each test sample was conducive for ensuring the reproducibility of results herein. **B.** The PDS was split into two test samples, given the differences in acquisition parameters and availability of data on cognition. The following demonstrations how many participants had a certain number of scan sessions: 3 had 1-session, 6 had 2-sessions, 18 had 3-sessions, 57 had 4-sessions, and 83 had 5-sessions.


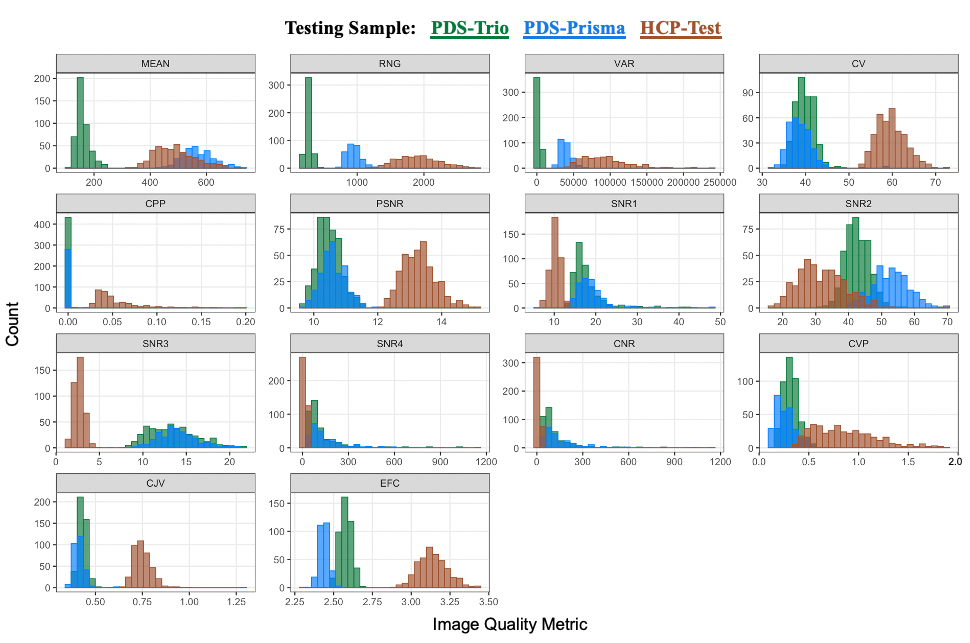


**Figure S2.** **Imaging acquisition differences between test samples resulted in varying levels of image quality across every metric.** One-way ANOVAs suggested that the three test samples differed across all quality metrics that were computed by the MRQy software package (Table 1). As detailed in the MRQy report^16^, abbreviations are defined as follows: MEAN: mean of foreground intensity values. RNG: range of foreground intensity values. VAR: variance of foreground intensity values. CV: coefficient of variation percentage. CPP: contrast per pixel. PSNR: peak signal-to-nose ratio. SNR: signal-to-noise ratio (4 variations). CNR: contrast-to-noise ratio. CVP: coefficient of variation of the foreground patch. CJV: coefficient of joint variation. EFC: entropy focus criterion.


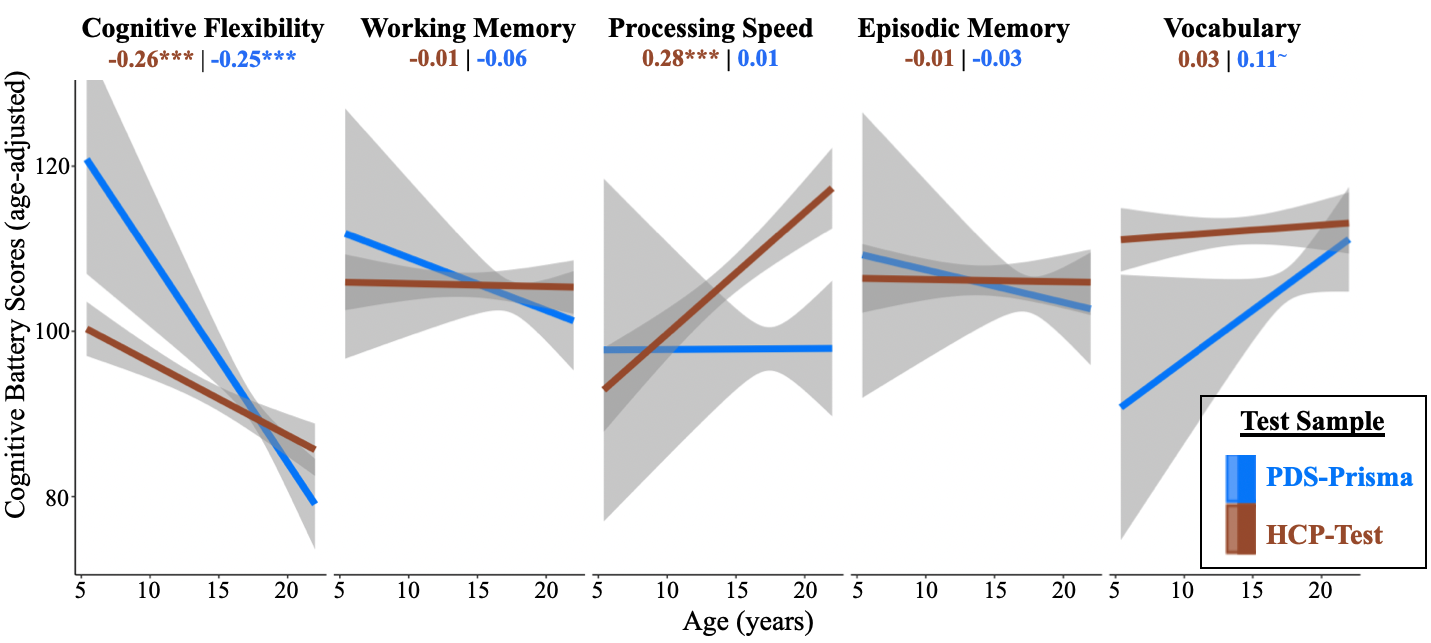
**Figure S3. Age-adjusted cognitive battery scores mostly did not vary as a function of age.** Of the 10 relationships analyzed only 2 were negatively correlated and 1 was positively associated. These findings provide reassurance that the significant relationships regarding model sensitivity, were not likely to be driven by age-related differences, despite that most brain age gaps from most variants contained linear age-related biases.
